# Myricetin: A Dietary Molecule with Diverse Biological Activities

**DOI:** 10.3390/nu8020090

**Published:** 2016-02-16

**Authors:** Deepak Kumar Semwal, Ruchi Badoni Semwal, Sandra Combrinck, Alvaro Viljoen

**Affiliations:** 1 Department of Pharmaceutical Sciences, Tshwane University of Technology, Pretoria 0001, South Africa; SemwalDK@tut.ac.za (D.K.S.); SemwalRB@tut.ac.za (R.B.S.); CombrinckS@tut.ac.za (S.C.); 2 SAMRC Herbal Drugs Research Unit, Faculty of Science, Tshwane University of Technology, Pretoria 0001, South Africa

**Keywords:** myricetin, anti-oxidant activity, anti-HIV activity, cytotoxicity, polyphenol, anti-alzheimer activity

## Abstract

Myricetin is a common plant-derived flavonoid and is well recognised for its nutraceuticals value. It is one of the key ingredients of various foods and beverages. The compound exhibits a wide range of activities that include strong anti-oxidant, anticancer, antidiabetic and anti-inflammatory activities. It displays several activities that are related to the central nervous system and numerous studies have suggested that the compound may be beneficial to protect against diseases such as Parkinson’s and Alzheimer’s. The use of myricetin as a preserving agent to extend the shelf life of foods containing oils and fats is attributed to the compound’s ability to protect lipids against oxidation. A detailed search of existing literature revealed that there is currently no comprehensive review available on this important molecule. Hence, the present work includes the history, synthesis, pharmaceutical applications and toxicity studies of myricetin. This report also highlights structure-activity relationships and mechanisms of action for various biological activities.

## 1. Introduction

Although myricetin occurs throughout the Plant Kingdom, it is produced mainly by members of the families Myricaceae [1,2], Anacardiaceae [3], Polygonaceae [4], Pinaceae [5] and Primulaceae [6]. This phenolic compound is very common in berries, vegetables, and in teas and wines produced from various plants. It occurs in both the free and glycosidically-bound forms, which include myricetin-3-*O*-(3″-acetyl)-α-l-arabinopyranoside, myricetin-3-*O*-(4″-acetyl)-α-l-arabinopyranoside, myricetin-3-*O*-α-l-rhamnopyranoside, myricetin-3-*O*-β-d-galactopyranoside, myricetin-3-*O*- (6″-galloyl)-β-d-galactopyranoside, myricetin-3-*O*-β-d-xylopyranoside, myricetin 3-*O*-α-l- arabinofuranoside [7], myricetin-3-*O*-(2”-*O*-galloyl)-α-l-rhamnoside, myricetin-3-*O*-(3”-*O*-galloyl)- α-l-rhamnoside and myricetin-3-*O*-α-l-rhamnoside [8]. Myricetin is poorly soluble in water, *i.e.*, 16.6 µg/mL, but dissolves rapidly when deprotonated in basic aqueous media and in some organic solvents such as dimethylformamide, dimethylacetamide, tetrahydrofuran and acetone [9]. Moreover, degradation of this compound, which is most stable at pH 2, was reported to be both pH and temperature dependent.

The history of myricetin (1) extends back to more than a hundred years. It was first isolated in the late eighteenth century from the bark of *Myrica nagi* Thunb. (Myricaceae), harvested in India, as light yellow-coloured crystals [10]. Isolation was primarily sparked by interest in the dyeing property of the compound. It was well characterised in a further study of Perkin [11], who established the melting point as 357 °C and prepared various bromo, methyl, ethyl and potassium analogues. This report also described myricitrin (2), a myricetin glycoside (myricetin-3-*O*-rhamnoside), for the first time. In a subsequent study, Perkin [12] found that myricetin yields a phloroglucinol and gallic acid upon hydrolysis, which served to confirm its chemical structure.

**Figure 1:**
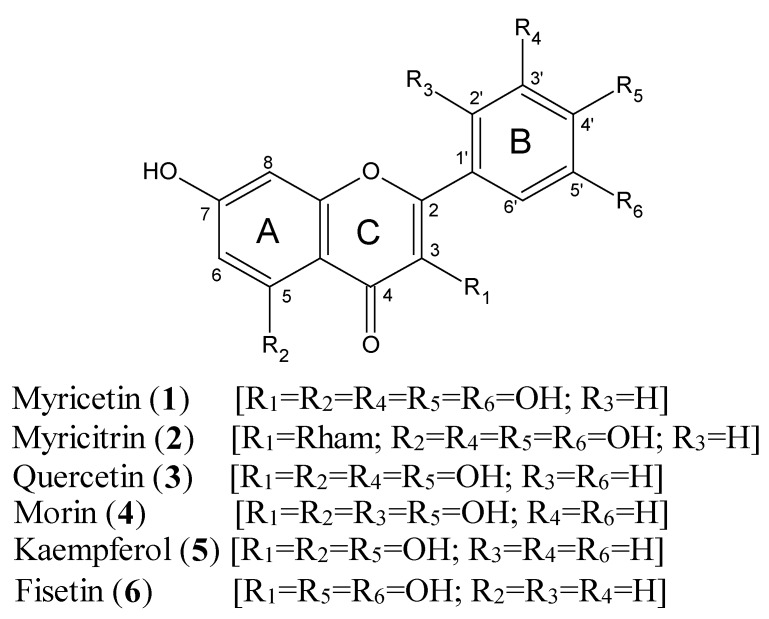
Chemical structures of myricetin and related compounds.

Myricetin (1) is structurally related to several well-known phenolic compounds (Figure 1), namely quercetin (3), morin (4), kaempferol (5) and fisetin (6). The compound is sometimes referred to as hydroxyquercetin, resulting from its structural similarity to quercetin (3). The nutraceuticals and anti-oxidant properties of myricetin are highly valued. Scientific evidence [13] underscores claims that the compound displays a variety of pharmacological activities, including anti-inflammatory, analgesic, antitumour, hepatoprotective and antidiabetic activities.

## 2. Chemical Synthesis

**Scheme 1:**
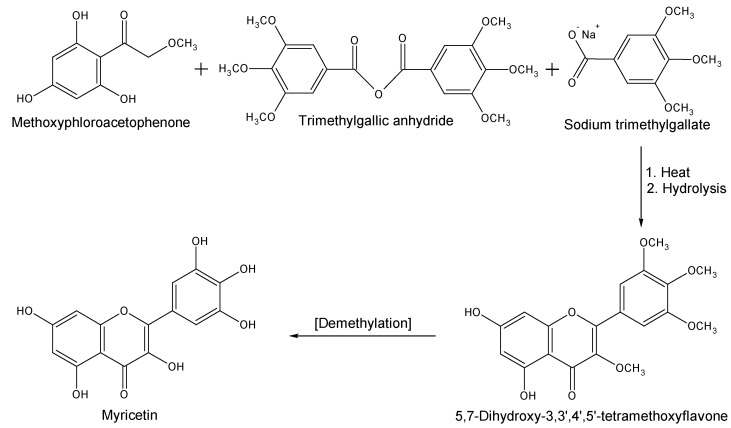
Synthesis of myricetin as proposed by Kalff and Robinson [17].

**Scheme 2:**
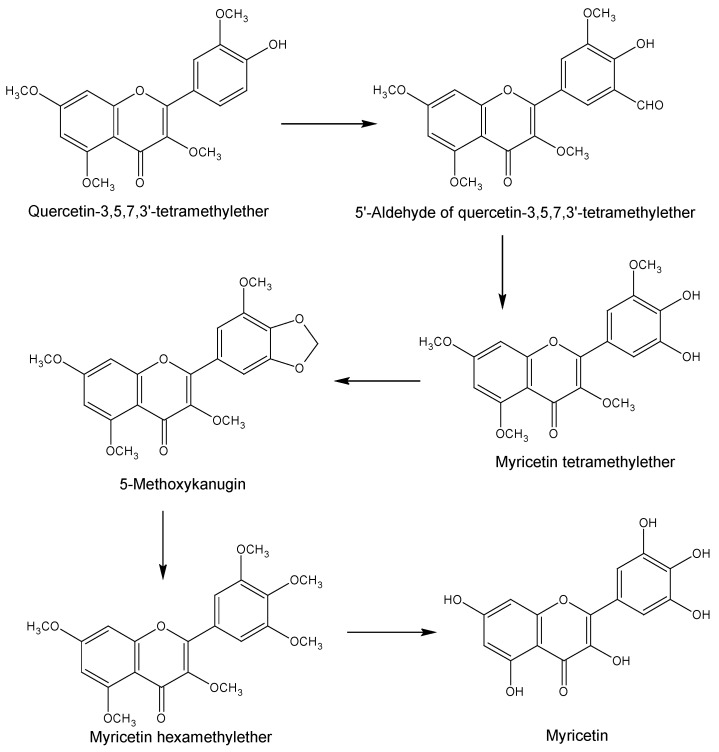
Route proposed by Rao and Seshadri [18] for the synthesis of myricetin.

The synthesis of myricetin is very important in terms of its use as a key starting material for the synthesis of various other beneficial compounds including hibiscetin [14,15]. Dean and Nierenstein [16] first attempted to synthesise myricetin in 1925 by applying the Kostanecki and Auwers procedure which was unfortunately, not successful. In the same year, Kalff and Robinson [17] managed to synthesise myricetin from ω-methoxyphloroacetophenone. This method involved heating the starting material together with trimethylgallic anhydride and sodium trimethylgallate. Following hydrolysis of the product, 5,7-dihydroxy-3,3′,4′,5′-tetramethoxyflavone was formed, which finally yielded myricetin after demethylation (Scheme 1). On the other hand, using an alternative route, Rao and Seshadri [18] synthesised myricetin from quercetin via an ortho-oxidation reaction (Scheme 2). In this procedure, 3,5,7,3′-tetra-*O*-methylquercetin was converted to the corresponding 5′-aldehyde, which was then converted to 3,5,7,3′-tetra-*O*-methylmyricetin to yield 5-methoxykanugin, following cyclisation at the 4′ and 5′ positions. Hydrolysis of 5-methoxykanugin, with subsequent methylation, yielded hexamethylmyricetin, which finally produced myricetin upon demethylation.

## 3. Pharmacological Applications

Myricetin is one of the key constituents of various human foods and beverages including vegetables, teas and fruits, and is recognised mainly for its iron-chelating, anti-oxidant, anti-inflammatory and anticancer properties [19]. Various studies have demonstrated its activity against a variety of DNA polymerases, RNA polymerases, reverse transcriptases, telomerases, kinases and helicases.

### 3.1. Anti-Oxidant Activity

An overwhelming body of information has been published concerning the anti-oxidant activity of myricetin, leaving no doubt that the compound is a powerful anti-oxidant. The results from Table 1 revealed that this compound exhibited the scavenging activity towards a number of radicals and ions.

**Table 1:** Scavenging activity of myricetin towards various radicals and ions. DPPH, 2,2-diphenyl-1-picrylhydrazyl; TEAC, Tetraethylammonium Chloride; ORAC, Oxygen Radical Absorbance Capacity; FRAP, Ferric Reducing Antioxidant Power; ROS, Reactive Oxygen Species; NO, Nitric Oxide.

Assay	Results	Control	Reference
DPPH	At 1 mg/mL inhibited DPPH radical by 71.5%. IC_50_ value was found to be 9 µg/mL	α-tocopherol (IC_50_ = 26 µg/mL) and BHT (IC_50_ = 30 µg/mL Trolox (1 mg/mL) inhibited DPPH radical by 61.5%	[20]
	At 0.01 mM (3.2 µg/mL), 0.1 mM (32 μg/mL) and 1 mM (320 µg/mL) inhibited DPPH radical by 85.6%, 92.8% and 96.9%, respectively, whereas IC_50_ value was 4 µM (1.3 µg/mL)	β-Actin as internal control	[21]
	At 40 µg/mL inhibited DPPH radical by 78%	Rutin (85% inhibition) at 40 µg/mL	[22]
Superoxide	Inhibited by 24.6%, 79.5% and 96.4% when applying concentrations of 0.001 mM (0.32 µg/mL), 0.01 mM (3.2 µg/mL) and 0.1 mM (32 µg/mL), respectively, while IC_50_ was calculated as 0.6 µM (0.2 µg/mL)	β-Actin as internal control	[21]
	At 1.86 μg/mL, scavenged superoxide radicals in the nitroblue tetrazolium hypoxanthine/xanthine oxidase assay	Ascorbic acid (IC_50_ 5.8 µg/mL)	[23]
TEAC	Activity of 2.40 mM (764 μg/mL) trolox/mg sample after 20 min. The IC_50_ value was found to 22 µg/mL	Trolox (0.2 mg/mL)	[24]
ORAC	1620 µmol trolox equivalent/g (515 mg/g)	-	[25]
FRAP	590 µmol Fe^2+^/L at 10 µM (0.32 μg/mL)	Gallic acid	[26]
Ascorbic acid-induced lipid peroxidation	Inhibited in rat brain by 92%, 95% and 95% at 0.1, 1.0 and 4.0 mM myricetin, respectively (concentrations correspond to 32, 320 μg/mL and 1.3 mg/mL, respectively).	Ascorbic acid (0.1 or 1.0 mM)	[27]
Ferrous sulfate-induced lipid peroxidation	Inhibited in rat brain by 28%, 71% and 91% at 0.1, 1.0 and 4.0 mM myricetin, respectively (concentrations correspond to 32, 320 μg/mL and 1.3 mg/mL, respectively).	Ferrous sulfate (1.0 mM)	[27]
Oleic acid triglyceride	Inhibited oleic acid-induced triglyceride over-accumulation towards HepG2 cells by 24.8% with IC_30_ > 150 µM (47 µg/mL)	-	[28]
ROS	34.5% inhibition with IC_30_ 122.7 µM (39.0 µg/mL)	-	[28]
NO	At a dosage of 50 mg/kg, decreased NO production by 56.7, 31.4, 7.7, 48.9 and 53.4 ng/g tissue in the brain cortex, liver, kidney, blood and lungs, respectively, of intact rats. Together with lipopolysaccharide (10 mg/kg) and at the same concentration, decreased the level of NO production in these organs by 206.5, 1008.3, 337.0, 542.8 and 824.8 ng/g tissue, respectively	-	[29]
	NO-scavenging capacity with k_AOx_/k_PTIO_ value of 1.2 TEU	Trolox (7.3 TEU)	[30]
Collagenase in human dermal fibroblasts	Inhibited by 12.7% and 29.6%.at myricetin concentration of 0.1 (32 μg/mL) and 0.2 mM (64 μg/mL), respectively	1,10-phenanthroline (39.4% and 75.1%, respectively)	[21]
Peroxynitrite anions	Antioxidant effects against peroxynitrite anions, chemiluminescence initiated by peroxynitrite in rat liver homogenate and lucigenin chemiluminescence in aortic rings with IC_50_ values of 35, 20 and 32 μM, respectively	-	[31]

The inhibition of ABTS^+^ and DPPH radicals by myricetin was found to be polyphenol oxidase-dependent [32]. However, Rusak and coworkers [33] reported that although the compound exerts a strong scavenging activity against DPPH radicals, it does not have activity against ROS in menadione-stressed HL-60 cells. The thiyl radical was reportedly inhibited by as much as 81.5% at a myricetin concentration of 500 µM (160 µg/mL). This radical serves as a catalyst for the *cis-trans* isomerization of fatty acids. It is generated from thiols and is induced by *trans*-arachidonic acid (TAA) formation during UV irradiation.

Myricetin was able to scavenge the hydroxyl free radicals generated through UV photolysis of H_2_O_2_ [34] and those formed in the mini-pig liver microsomal system [35], a doxorubicin-dependent process. It also displayed significant anti-oxidant activity against the peroxyl radical generated from 2,2′-azobis-(2-amidinopropane)-dihydrochloride [36]. The compound displayed poor activity (IC_50_ value = 1.4 mg/mL) in a superoxide dismutase (SOD)-like activity assay. It was found to protect cells against H_2_O_2_-induced cell damage via inhibition of ROS generation and activation of antioxidant enzymes [37]. Moreover, it prevented oxidative stress-induced apoptosis via regulation of PI3K/Akt and MAPK signalling pathways. The compound was found to restore the activity and protein expression of cellular anti-oxidant defence enzymes such as SOD, catalase (CAT), and glutathione peroxidase (GPx) reduced by H_2_O_2_ treatment [38].

Duthie and coworkers [39] reported that myricetin, at a concentration of 100 μM, restricts H_2_O_2_-induced DNA strand breakage in human lymphocytes, in the absence of genotoxicity. The same research group [40] proposed that the compound, at an effective concentration of 1 mM, protects against DNA strand breakage in human colonocyte Caco-2 cells resulting from oxidative attack caused by H_2_O_2_. Miyajima and coworkers [41] reported that it has an inhibitory effect on the peroxidation of liposomes. It induces the degradation of nuclear DNA that is concurrent with lipid peroxidation and is enhanced by Fe(III) or Cu(II) [42]. Myricetin-induced lipid peroxidation was inhibited by SOD in the presence of Cu(II), but was increased by CAT and sodium azide in the presence of Fe(III). The compound displayed cytoprotective effects against Fe(III)-induced genotoxicity via stimulation of DNA repair processes. Myricetin at 25, 50 and 100 μM, in the presence of Fe(III), prevented lipid peroxidation and stimulated the release of DNA oxidation bases into culture media [43]. A study by Morel and coworkers [44] revealed that myricetin at 300 μM is able to inhibit lipid peroxidation in Fe-treated rat hepatocyte cultures. At this concentration, phenoxyl radical intermediates are formed that possibly contribute to the mode of action.

The anti-oxidant property of myricetin was found to exceed that of Vitamin E (d-α-tocopherol) [45]. It reacted 28 times faster with oxygen-centred galvinoxyl radicals and reduced these radicals twice as fast as Vitamin E. However, the compound was unable to protect Vitamin E-deficient microsomes from lipid peroxidation. Myricetin (5 µM) potentially inhibited the SIN-1-mediated breakage of DNA strands, a process that results in the generation of equivalent amounts of NO and O_2_^−^ through autoxidation [46]. A significant decrease in the oxygen consumption resulting from 250 µM of SIN-1 was noted when increasing the myricetin concentration to 40 µM. The formation of peroxynitrite indicated that the autoxidation of SIN-1 had been restricted. In combination with Vitamins C and E, myricetin exerted anti-oxidant activity by modulating the anti-oxidant enzyme system and scavenging free radicals [47]. The combined treatment increased anti-oxidant enzyme CAT activity, while reducing SOD and glutathione peroxidase activities, as well as the ROS levels within B16F10 murine melanoma cells. Myricetin was also found to protect against ROS production in the red blood cells of normal individuals and in patients with sickle cell anemia at 30, 50 and 100 μM [48]. In addition, it exhibited anti-oxidant potential against superoxide anions generated in the xanthine-xanthine oxidase and phenazine methosulfate-NADH systems. It also inhibited malondialdehyde formation by rat liver microsomes [49,50].

A molecular mechanism-based study by Qin and coworkers [51] suggested that Nrf2-mediated anti-oxidant response element activation is involved in myricetin-induced expression profiling in hepatic HepG2 cells. They found that of a total of 44,000 gene probes in HepG2 cells, myricetin is able to upregulate the signals of 143 and downregulate 476 of them, twofold or more. At concentrations of 20, 40 and 60 μM, myricetin displayed better *in vitro* cytoprotective effects against H_2_O_2_ or CCl_4_-induced oxidative injury in human hepatocyte (HL-7702) cells than α-tocopherol (positive control). It also improved cell viability, increased reduced glutathione content in cells, reduced lactate dehydrogenase leakage into culture medium and decreased the formation of malondialdehyde in hepatocyte cells [52].

At a low concentration, *i.e.*, IC_50_ ≤ 1.5 μM, myricetin was found to be a potent inhibitor of Fe-induced lipid peroxidation in rat liver microsomes. However, at a higher concentration it displayed peroxidant effects against hydroxy radicals. At 100 μM, the compound enhanced hydroxy radical formation from H_2_O_2_ up to eight fold, in the presence of Fe^3+^-EDTA. Myricetin, in the presence of the antitumour antibiotic and antiviral drug, bleomycin, caused damage to DNA. It also accelerated bleomycin-dependent DNA damage at 75 μM, most likely by reducing the Fe^3+^-bleomycin-DNA complex to the Fe^2+^ form. These findings imply that myricetin acts as an anti-oxidant only at lower concentrations, while at higher concentrations it has pro-oxidant effects [53]. Chobot and Hadacek [54] later supported this deduction by demonstrating that myricetin exhibits both anti-oxidant and pro-oxidant effects by using two different variants, *i.e.*, FeCl_3_ and Fe-EDTA. Their results revealed that in the presence of ascorbic acid, the compound has an anti-oxidant effect when complexed with Fe^3+^. This activity depends on ROS scavenging and the chelation properties of Fe^3+^ ions. However, in ascorbic acid-free systems, the pro-oxidant effects were enhanced when Fe^3+^ was complexed with EDTA, which may be caused by the reduction of molecular oxygen to ROS and Fe^3+^ to Fe^2+^. Myricetin was found to delay the onset of ascorbate/Fe^2+^/ADP complex-initiated peroxidation (estimated by the formation of TBARS) in hepatic microsomal preparations of Vitamin E-deficient rats by 12.6 and 14.3 min at concentrations of 0.25 and 0.5 mM, respectively. However, at the same concentration, the lag phase of Vitamin E, the positive control, was greater than 20 min [55].

Myricetin proved to inhibit the *tert*-butylhydroperoxide (*t*-BOOH)-initiated chemiluminescence of mouse liver homogenates as reflected by the obtained IC_50_ value of 15 mM [56]. These results suggest that the compound may have potential to protect against lipid peroxidation and other free radical-mediated cell injuries. The compound also mitigated *t*-BOOH-induced increases in the levels of oxidative stress parameters including malondialdehyde and the protein carbonyl group of erythrocytes from Type-2 diabetic patients *in vitro* [57]. These findings suggest that supplementation of the diet with myricetin or myricetin-rich food may be beneficial to all pathological conditions where the anti-oxidant system of the body is overwhelmed.

At a concentration above or equal to 3 µM, incubation with myricetin reduced the oxidative metabolism of 2′,7′-dichlorofluorescin (DCFH) in the resting brain neurons of rats. The fluorescent dye is retained within the neurons and oxidized by cellular hydrogen peroxide to a substance that is highly fluorescent. Moreover, myricetin reduced the Ca^2+^-induced increase in oxidative metabolism, without affecting the cellular content of DCFH or the intracellular concentrations of Ca^2+^. This anti-oxidant effect may be responsible for the positive effects on brain neurons subjected to ischemia [58].

Since the peroxyl radical-scavenging activities of test substances have been found to depend upon the number of hydroxy substituents present, myricetin with its six hydroxy moieties could be expected to have a strong radical absorbing capability in the 2,2′-azobis-(2-amidinopropane)-dihydrochloride assay [36]. Teissedre and coworkers [59] found that the inhibition of LDL oxidation by myricetin and other compounds was dependent on the number of hydroxy groups in ring-B. Myricetin was able to inhibit LDL oxidation by 68.1%, 97.4% and 97.6% at 5, 10 and 20 µM gallic acid molecular weight equivalents, respectively. A mechanism-based study [60] also indicated that the anti-oxidant activity of myricetin mainly depends on the B-ring. A similar study by Xie and coworkers [61] revealed that the hydroxy group in the C-4′ position plays the biggest role in the activity of myricetin against lipid peroxide radical CH_3_OO∙. The higher activity of the 4′-hydroxy site is perhaps due to weak H-bonding interactions between the oxygen radical of the reactive hydroxy group and the adjacent hydroxy group in the B-ring. Analysis of structure-activity relationships suggested that the free radical scavenging activity of myricetin varies, depending on the type of free radical. The presence of the 3′,4′-catechol moiety in the B-ring was linked to a strong DPPH scavenging activity, whereas the hydroxy moiety at position C-4′ was thought to be responsible for the reduction of xanthing/xanthine oxidase-generated superoxide. However, any additional hydroxyl group at position 3′ or 5′ was found to reduce anti-oxidant potency [62]. The reducing properties of myricetin towards Cu and Fe ions were attributed to the double bond at the C2-C3 position, the catechol in ring-B and the 3-hydroxy functional groups [63,64].

### 3.2. Antiphotoaging Activity

Myricetin exhibited antiphotoaging effects by quenching causative free radicals in the skin. Topical application of the compound was found to inhibit chronic ultraviolet-B (UVB) irradiation-induced wrinkles in mouse skin, by suppressing UVB-induced Raf-kinase activity and subsequent attenuation of phosphorylation of MEK and ERK. It also reduced epidermal thickening resulting from UVB irradiation and suppressed matrix metalloproteinase-9 (MMP-9) protein expression and enzyme activity in mice [65]. A mechanism-based study revealed that myricetin attenuates UVB-induced keratinocyte death and reduces malondialdehyde levels, which are increased following exposure to UVB rays. The ability of myricetin to inhibit UVB-induced generation of H_2_O_2_ in keratinocytes can be linked to its anti-oxidant potential, which promotes the scavenging of free radicals. The compound also inhibits the UVB-induced activation of c-jun-NH_2_ terminal kinase (JNK) in keratinocytes [66]. Myricetin is able to suppress UVB-induced COX-2 expression in mouse skin epidermal JB6 P+ cells. It inhibits UVB-induced initiation of activator protein-1 and NF-κβ, as well as Fyn kinase activity. This activity was found to be similar to that of 4-amino-5-(4-chloro-phenyl)-7-(*t*-butyl)-pyrazolo[3,4-d]pyrimidine, a prominent Fyn inhibitor. Furthermore, the compound inhibits MEK1 kinase activity and transformation of JB6 P+ mouse epidermal cells *in vitro* [67]. Kim and coworkers [68] found that myricetin can reduce Akt activity and stimulate apoptosis in UVB-irradiated keratinocyte HaCaT cells by decreasing phosphorylation of Akt and Bad (a pro-apoptotic protein) at a concentration of 20 μM. Moreover, Kumamoto and coworkers [69] reported that myricetin may perhaps directly target Akt to restrict cell transformation. Both *in vitro* and *ex vivo* models revealed that the compound is able to inhibit the phosphorylation and kinase activity of Akt.

### 3.3. Anticancer Activity

Extensive research into the anticancer activities of myricetin has indicated that the compound is cytotoxic towards a number of human cancer cell lines, including hepatic, skin, pancreatic and colon cancer cells (Table 2). It also inhibits key enzymes involved in the initiation and progression of cancer.

Myricetin possesses anti-proliferative activity against human acute leukemia HL-60 cells; the activity was enhanced with increasing concentration [70]. A study of the mode of action revealed that the C2-C3-double bond, aromatic ring-B at C-2 and hydroxy groups in ring-B are possibly responsible for the cytotoxicity. The compound displayed cytotoxicity towards chronic myeloid human leukemia K562 cells and normal peripheral blood mononuclear cells isolated from the blood of a healthy human [71].

Myricetin also stimulated high concentrations of topo-DNA complexes with topoisomerase (topo) I and topo II enzymes in K562 cells. Notably, mice embryo fibroblasts lacking topo IIβ were found resistant to cell-growth inhibition induced by myricetin, which suggests that a specific concentration of myricetin is required to produce topo-mediated carcinogenic and chemotherapeutic effects in an *in vivo* system [72]. The structure-activity relationship analysis related to the inhibition of topo I and II suggested that hydroxy group substitution at C-3, 7, 3′ 4′, a carbonyl moiety at C-4 and saturation at C2-C3 are prerequisites for this activity [73]. An additional hydroxyl group in ring-B was found to further enhance the topo I inhibitory activity.

Myricetin was found to protect against skin cancer, by strongly inhibiting tumour promoter-induced neoplastic cell transformation through restriction of MEK, JAK1, Akt and MKK4 kinase activity [74]. At a concentration of 10 μM, the compound inhibited TPA- and EGF-induced cell transformation by 76% and 72%, respectively. Its combination with resveratrol it produced an additive, but not a synergistic effect, towards either TPA- or EGF-induced transformation. Myricetin also attenuated tumour promoter-induced activation of c-fos or activator protein-1 [75]. It was found capable of inhibiting JAK1/STAT3 pathways, thereby blocking cell transformation in EGF-activated mouse JB6 P+ cells. The compound was found to not only inhibit DNA-binding and transcriptional activity of STAT3, but also the phosphorylation of STAT3 at Tyr705 and Ser727 [76]. Ichimatsu and coworkers [77] earlier reported that the compound caused marked inhibition of EGF-induced transformation of mouse epidermal cells. Suppression of the EGF-induced trigger for activator protein-1 was achieved. The presence of the 3-, 3′- and 4′-hydroxy groups, C2-C3 double bond and the phenylchromone skeleton were found to be responsible for the activity.

**Table 2:** Anticancer activity of myricetin towards various cancer cell lines.

Cell line/Enzyme	Effect of Myricetin	Reference
**Activity on Cell Lines**		
**Brain**		
U251, NCH89 and LN229 cells	No effect when alone, since the IC_50_ value for each cell line was found to be >200 µM. A combination of myricetin (150 µM) and TRAIL (50 ng/mL) yielded a synergistic activity and increased cell death in U251, NCH89 and LN229 by 59%, 65% and 52%, respectively.	[78]
**Breast**		
MCF-7	IC_50_ 2.70 μg/mL compared to vinblastine (IC_50_ 45.6 μg/mL)	[79]
	Increased GSH content of cells and also increased the EROD reaction 2-fold at a concentration of 25 μM	[80]
**Cervix**		
HeLa cells	Cytotoxic with IC_50_ 18.9 µg/mL	[81]
**Colon**		
Epithelial adenocarcinoma cells	Proliferation of cells inhibited at 50 μM by decreasing COX-2 and cyclin D1 expression	[82]
HCT116	Inhibited the proliferation of human colon carcinoma cells by halting the cell cycle in G2/M phase and inducing apoptosis; LD_50_ 28.2 μM	[83]
COLO 205, COLO 320HSR, COLO 320DM, HT 29 and COLO 205-X	Inhibited the activation of MMP-2 enzyme in the cells with IC_50_ values of 7.82, 11.18, 11.56, 13.25 and 23.51 μM, respectively. It also suppressed TPA-induced MMP-2 protein expression in COLO 205 cells by blocking the translocation of PKCα from cytosol to membrane, phosphorylation of ERK1/2 protein and induction of c-Jun protein expression activated by TPA.	[84]
**Leukemia**		
HL-60	Alone, and in combination with piceatannol, induced apoptotic cell death through a ROS-independent cell death pathway. The effect was greater with the combined treatment	[85]
	Anti-proliferative activity and the effect was enhanced with increasing concentration	[70]
**Prostate**		
LNCaP	IC_50_ value 2.10 μg/mL while taxol (IC_50_ 0.08 μg/mL) used as standard	[79]
22Rv1	Inhibition of TCDD-induced EROD activity in cancer cells; IC_50_ value 3.0 μM	[86]
**Uterus**		
RL95-2 endometrial cancer cells	Inhibition of CYP1 activity of cancer cells; IC_50_ values 3 μM and lower	[87]
**Inhibition of enzyme/protein activity**		
Thioredoxin reductase (TrxR) from mammals	Inhibitory effect on enzyme, which is overexpressed in many aggressive tumours; IC_50_ value 0.62 μM. Attacks the reduced COOH-terminal of -Cys-Sec-Gly, the active site of TrxR	[88]
TrxR	At 50 μM, inhibited growth of A549 (human lung carcinoma) cells and reduced TrxR activity in the cell lysates, corresponding with the oxidization of thioredoxin	[88]
Mammalian DNA polymerases	IC_50_ values ranged from 21.3 to 40.9 μM. Human DNA topoisomerase II activity inhibited; IC_50_ 27.5 μM	[83]
Phosphatidylinositol 3-kinase (PI3K)	Inhibited this enzyme (IC_50_ 1.8 μM) that plays an important role in signal transduction and cell transformation. Also inhibited PKC and tyrosine kinase activity of EGF-R	[89,90]
E6, a primary oncoprotein of human papillomaviruses	Inhibited E6, responsible for cervical cancer by inhibiting GST-E6 and His-caspase 8 binding	[91]
CCAAT-enhancer-binding proteins-α, peroxisome proliferator-activated receptor-γ, lipoprotein lipase, fatty acid binding aP2 protein and adiponectin	At 30 μM, myricetin decreased mRNA levels of these enzymes. Inhibited adipogenesis in human adipose tissue-derived mesenchymal stem cells.	[92]
Multidrug resistance-associated protein MRP1 and MRP2 mediated vincristine efflux in MDCKII cells	Inhibitory effects with IC_50_ 30.5 and 24.6 μM, respectively. At a concentration of 25 μM, it increased the sensitivity of the cells towards vincristine toxicity towards MRP1 and MRP2 cells with IC_50_ values of 7.6 and 5.8 μM, respectively	[93]

Although myricetin displayed moderate cytotoxicity towards human laryngeal carcinoma HEp2 cells, its activity against their drug-resistant CK2 subline was relatively poor [94]. The compound increased the expression of cytochrome CYP1A1 in both cell lines. A mechanism-based study by Xu and coworkers [95] revealed that myricetin exerts strong inhibitory activity against human prostate cancer PC-3 cells. The effect was found to increase with increasing concentrations of the compound, up to 300 μM. Moreover, combination with myricitrin produced a strong synergistic effect, resulting in a decrease in cell proliferation. Similarly, myricetin alone, or in combination with myricetrin, also induced PC-3 cell apoptosis, which was further enhanced with increasing concentration.

The viability and proliferation of bladder cancer T24 cells was decreased following exposure to myricetin, while the migration of T24 cells was decreased through a reduction in *in vitro* MMP-9 expression [96]. Moreover, the compound induced apoptosis and promoted cell cycle arrest at G2/M by downregulating cyclin B1 and cyclin-dependent kinase cdc2. The mode of action suggests that myricetin inhibits the phosphorylation of Akt, while increasing the phosphorylation of p38 MAPK. Proliferation of human hepatoma cancer HepG2 cells was decreased and G2/M phase arrest was induced by the compound. It increased protein levels in the p53/p21 cascade, while decreasing Cdc2 and cyclin B1 protein levels in HepG2 cells [97]. Moreover, the upregulation of Thr14/Tyr15 phosphorylated Cdc2 and p27, and the downregulation of CDK7 kinase protein and CDK7-mediated Thr161 phosphorylated Cdc2 were recorded after treatment with myricetin.

The compound was found to exert moderate cytotoxicity, which was mediated by G2/M cell cycle arrest and apoptosis, towards human oesophageal adenocarcinoma OE33 cells [98]. Investigation of the mechanism revealed that G2/M cell cycle arrest by myricetin occurs via up-regulation of GADD45β and 14-3-3σ and down-regulation of cyclin B1 at the mRNA and protein levels. Myricetin was reported to stimulate the expression of PIG3 mRNA and the protein levels in human oesophageal cancer KYSE-510 and OE33 cells [99,100]. Induction of PIG3 caused apoptosis in cancer cells through the mitochondrial pathway in a p53-independent manner [101]. It provided significant anti-proliferative effects against melanocyte B16F10, SK-MEL-1 and Melan-A cells. The C2-C3 double bond and hydroxy substituents in myricetin were found to be responsible for the activity. The inhibition of activity of phosphatidylinositol 3-kinase (PI3K), an enzyme that plays an important role in signal transduction and cell transformation, and the reduction of PKC and tyrosine kinase activity of EGF-R were also attributed to the hydroxy moieties in ring-B and the C2-C3 double bond [89,90].

Myricetin was also found to be active against medulloblastoma, a malignant brain tumour that commonly occurs in children. It inhibited HGF/Met signalling in medulloblastoma DAOY cells and prevented the formation of actin-rich membrane ruffles [102].

The compound caused metastasis of human lung carcinoma A549 cells *in vitro* by limiting the adhesion, invasion and migration of cancer cells without showing cytotoxicity against normal cells [103]. Myricetin was able to inhibit MMP-2, urokinaseplasminogen activator activities, phosphorylation of ERK1/2 and the activation of NF-kβ, c-Fos and c-Jun. It exhibited *in vitro* activity against primary and metastatic pancreatic cancer cells by inducing cancer cell death via apoptosis, while decreasing PI3 kinase activity [104]. Moreover, an *in vivo* treatment resulted in tumour regression and a reduction in the metastatic spread of orthotopic pancreatic tumours. Myricetin was found to be non-toxic towards normal cells.

The compound can be regarded as a potent chemoprotective agent against prostate cancer [105]. It was found to induce cytotoxicity and DNA condensation in human colon cancer HCT-15 cells, and increased the BCL2-associated X protein/B-cell lymphoma 2 ratio in cancer cells [106]. A significant surge in the release of apoptosis-inducing factor from mitochondria was recorded in the presence of myricetin. The compound stimulated the basolateral uptake of the pro-carcinogen 2-amino-1-methyl-6-phenylimidazo[4,5-b]pyridine (PhIP), by partially inhibiting the MRP2-mediated excretion of PhIP from intestinal cells back to the lumen [107].

Myricetin, on the other hand, in combination with the food mutagens, 3-amino-1-methyl-5H-pyrido-(4,3-b)indole (Trp) and 2-amino-3-methylimidazo-(4,5-f) quinoline (IQ), displayed antigenotoxic effects in human lymphocytes and reduced DNA damage in the absence of exogenous metabolic activation [108].

### 3.4. Anti-Platelet Aggregation Activity

Exposure to 150 µM myricetin caused 14%, 26%, 5% and 49% inhibition of rabbit platelet aggregation, induced by ADP, arachidonic acid, collagen and PAF, respectively [109]. The compound suppressed thromboxane B2 formation in platelets challenged with arachidonic acid. Their study revealed that the antiplatelet activity of myricetin may be due to the inhibition of thromboxane formation. Zang and coworkers [110] found that myricetin inhibited specific receptor binding of platelet activating factor (PAF) in rabbit platelets. They reported IC_50_ values of 34.8, 85.7 and 119 μM for [3H] PAF at myricetin concentrations of 1, 2 and 4 nM, respectively. In addition, an IC_50_ of 13.1 μM of myricetin was established for the inhibition of PAF-induced reactions involved in rabbit platelet adhesion.

Moreover, the compound was found to be active against thrombin and neutrophil elastase with IC_50_ values of 28 and 7 µM, respectively [111]. Its ability to inhibit rabbit platelet aggregation and PAF-induced 5-HT release is reflected by the respective IC_50_ values of 17.5 and 64.1 μM obtained. However, the compound at a concentration of 7.9 μM had no effect against 5-HT release from platelets [112]. A docking experiment indicated that myricetin has the potential to inhibit thrombin and that the compound could therefore be helpful in the treatment of thrombotic disease [113].

A prostacyclin-stimulated rise in the levels of platelet adenosine 3′,5′-cyclic monophosphate (cyclic-AMP) was stimulated by myricetin. The mechanism of anti-aggregating activity revealed that modification of platelet cyclic-AMP metabolism occurred via inhibition of phosphodiesterase activity [114]. An intravenous dose of myricetin at 3.6 µg/kg body weight inhibited platelet aggregation in cat blood. However, in an *in vitro* experiment, it was found to disaggregate platelet thrombi at a concentration of 60 nM. The compound was shown to bind platelet membranes and prevent prostacyclin synthase against oxygen radicals such as superoxide anion radical (O_2_∙^−^), singlet oxygen, hydroxyl radical (∙OH) and perhydroxyl radical (HO_2_) because of its anti-oxidant effect [115]. Myricetin lowered the content of PGE2 in peritoneal fluid and reduced platelet aggregation induced by collagen and arachidonic acid *in vitro* [116]. The study suggested that the compound is a potent COX-1 inhibitor with anti-platelet effects.

### 3.5. Antihypertensive Activity

The antihypertensive activity of myricetin has been demonstrated *in vivo*. Hypertension and oxidative stress initiated by deoxycorticosterone acetate (DOCA) was reduced after treatment with oral doses of 100 and 300 mg myricetin/kg body weight in rats [117]. A reduction in systolic blood pressure, changes in vascular reactivity and a reversal of DOCA-induced increase in heart rate was evident. In addition, it reversed increasing levels of thiobarbituric acid-reactive substances and decreasing levels of SOD and CAT, and also reduced glutathione concentrations in the heart tissue of rats after exposure to DOCA. This study confirmed the results of an earlier six weeks *in vivo* study by Godse and coworkers [118] which revealed that myricetin reduces systolic blood pressure and vascular reactivity changes to catecholamines. Myricetin lowered high blood pressure induced by a diet rich in fructose at doses of 100 and 300 mg/kg p.o. in rats and reversed metabolic alterations stimulated by the sugar.

### 3.6. Immunomodulatory Activity

Various *in vitro* and *in vivo* researches revealed that myricetin has capability to modify the immune response or functioning of the immune system by stimulating antibody formation or inhibiting the activity of WBCs in the experiment model. It was found to modulate LPS-stimulated activation of mouse bone marrow-derived dendritic cells (DCs) without exhibiting any toxic effects towards DCs at a concentration of 10 μg/mL. The secretion of TNF-α, IL-6 and IL-12 in LPS-stimulated DCs was decreased by exposure to myricetin. The compound also inhibited the expression of LPS-induced major histocompatibility class II, CD40 and CD86 on DCs, while blocking the endocytic and migratory capacity of LPS-stimulated DCs. Moreover, it was found to suppress LPS-induced lymphocyte proliferation at a concentration of 10^−^^5^ M. A study by Kang and coworkers [119] to elucidate the mode of action suggested that myricetin inhibits LPS-induced IL-12 production in mouse primary macrophages and in the RAW264.7 monocytic cell-line, via the downregulation of NF-κB binding activity. The compound at 50 μM induces endothelium-dependent contractile responses in isolated rat aortic rings and stimulates the production of cytosolic free calcium in cultured bovine endothelial cells [120].

Myricetin exerted immunosuppressive effects by inhibiting the secretion of IL-2 protein from mouse EL-4 T cells, activated with PMA plus Io. In addition, it suppresses the intracellular production of the IL-2 protein, and reduced the expression of IL-2 mRNA induced by PMA plus Io [121]. Moreover, at concentrations of 5–100 μM, the compound inhibited CD69 expression of mouse CD_3_+T cell and proliferation of mouse lymphocytes *in vitro*. A complete arrest of IL-2 mRNA expression by mouse lymphocytes resulted from treatment with 100 μM myricetin [122]. On the bases of above findings, it can be corroborated that myricetin has potential to modulate the immune system. However, further researches are needed to develop it as an immunomodulatory drug.

### 3.7. Anti-Inflammatory Activity

The anti-inflammatory activity of myricetin has been demonstrated in a variety of *in vitro* assays, as well as in both acute and chronic *in vivo* animal models [38]. Myricetin (62.5–125 μg/mL) showed activity against the *Porphyromonas gingivalis*-induced inflammatory response in host cells and prevented NF-κB activation in a monocyte model. In addition, this molecule inhibits the secretion of IL-6, IL-8 and MMP-3 by *P. gingivalis*-stimulated gingival fibroblasts. The study suggested that myricetin can act as a therapeutic agent for the treatment of periodontitis, a serious gum infection that damages the soft tissue and destroys the bone that supports your teeth [123].

Myricetin displayed anti-inflammatory activity by inhibiting the production of LPS-induced prostaglandins [124]. The structure-activity relationship suggested that the double-bond at C2-C3 and keto group at C-4 are the most likely factors responsible for the strong inhibitory effect towards COX-2 expression. At a concentration of 10 μM, myricetin inhibited NO production in endotoxin-stimulated RAW264.7 murine macrophages, without cytotoxicity being evident [62]. The compound was also found to inhibit the production of LPS-stimulated NO, pro-inflammatory cytokines, PGE2 production and protein levels of iNOS and COX-2 in RAW 264.7 macrophages [125]. A study by Lee and coworkers [126] using JB6 P+ mouse epidermal cells revealed that myricetin inhibits phorbol ester-induced COX-2 expression by suppressing activation of NF-κB at concentrations of 10 and 20 μM. It also attenuated the phorbol ester-induced production of PGE2 and blocked the phorbol ester-stimulated DNA binding activity of NF-κB.

Myricetin was found to be active against periodontitis, an infectious inflammatory disease caused by microbes of dental bacterial plaque that affect the connective tissue and supporting bone surrounding the teeth. Activation of ERK-1/2, AKT and p38, and lipoteichoic acid-induced COX-2 expression in human gingival fibroblasts was inhibited by the compound. It also blocked IκB degradation and PGE2 synthesis and expression [127,128]. Myricetin did not have any effect on cell viability, but decreased the mRNA expression and enzyme activity of MMP-1, -2 and -8 in human growth factor (HGF). This compound also inhibited the RANKL-stimulated activation of p-38, ERK and cSrc signalling, and the RANKL-stimulated degradation of IkB in RAW264.7 cells. Moreover, the secretion of LPS-induced TNF-α and IL-1β in RAW264.7 cells was significantly inhibited by myricetin [129].

The compound displayed activity against rheumatoid arthritis by inducing differentiation in human MG-63 osteoblast-like cells at various concentrations, *i.e.*, 1, 5, 10 and 20 µM, in the absence of cytotoxicity against the MG-63 cell viability. It also inhibited anti-Fas IgM-induced apoptosis and blocked the synergetic effect of anti-Fas IgM with TNF-α or IL-1β on cell death in MG-63 cells [130]. Moreover, it exhibited anti-arthritic activity by reducing IL-1β-induced production of MMP and IL-6 in SW982 synovial cells and also by inhibiting JNK and p38 MAPK [131].

Myricetin inhibited xylene-induced ear oedema and carrageenan-induced hind paw oedema, and also restricted acetic acid-induced vascular permeability in the human body. The compound reduced serum levels of malonyl dialdehyde, while increasing the serum levels of SOD in the carrageenan-induced paw oedema model. The study suggested that the anti-inflammatory activity of this polyphenolic molecule might be linked to its anti-oxidant effect. Oral administration of myricetin at 100 and 200 mg/kg, b.w. ameliorated body weight loss and reduced histology scores, following dextran sulfate sodium-induced acute experimental colitis in mice [132]. It decreased the concentrations of IL-1β and IL-6, as well as the production of NO, myeloperoxidase and malondialdehyde. However, the activities of SOD and GSH-Px were significantly increased by myricetin. By acting as a co-substrate for the cyclo-oxygenases, the compound stimulated the formation of prostaglandin products in Sprague-Dawley rats [133]. It was observed that myricetin doses below 0.3 mg/kg b.w. stimulate the formation of PGE2, while higher doses lower the stimulatory effect.

### 3.8. Anti-Allergic Activity

Myricetin was reported to have an anti-allergic effect in a murine model of OVA-induced allergy. Oral administration of myricetin (5 mg/kg) to OVA-sensitized BALB/c mice inhibited pulmonary cell migration and IgE and IgG1 OVA-specific production [134]. Mast cell-mediated allergic inflammation was significantly reduced by exposure to myricetin, through inhibition of IgE or PMACI-mediated histamine release in RBL-2H3 cells [135]. The compound inhibited the elevation of intracellular calcium, and attenuated TNF-α and IL-6.

### 3.9. Analgesic Activity

Myricetin (0.1–10 mg/kg *i.p.*) produced a remarkable analgesic effect in a neuropathic pain model in rats, by reducing spinal nerve ligation-induced mechanical allodynia and thermal hyperalgesia lasting for several hours [136]. It was reported that the compound reduced voltage-activated calcium channel currents (ICa(V)) *in vitro* by 10%–56% at lower concentrations (0.1–5 μM), whereas at higher concentrations (10–100 μM), it stimulated a 20%–40% increase in ICa(V). The mechanism of action revealed that the analgesic activity of myricetin could be related to its PKC-induced decrease of ICa(V) in rat dorsal root ganglia neurons. Hagenacker and coworkers [137] reported that the compound also reduced voltage-activated potassium channel currents (IK(V)) *in vitro* by 18%–78% at concentrations of 1–75 μM, but the results were independent of the voltage applied. This reduction of IK(V) in rat sensory neurons was found to be p38 dependent. Myricetin exerted a significant analgesic effect, as reflected by the acetic acid-induced writhing response and the licking time in the late phase of the formalin test [116]. Its analgesic activity was found to be unrelated to sedation, because myricetin was unable to increase the pentobarbital-induced sleep time. The compound was active at doses of 10–100 mg/kg *i.p.* in the bradykinin-induced nociception assay, manifested in the hind paws of mice. The ID_50_ value was measured as 12.4 mg/kg. At a dose of 100 mg/kg *i.p.*, the compound also reduced cinnamaldehyde-induced nociception by 57%. Acidified saline-induced nociceptive responses were significantly inhibited by myricetin at doses of 30–100 mg/kg, *i.p.* [138]. An inhibition of 71% was recorded with an ID_50_ value of 22 mg/kg. Similarly, at doses of 10–30 mg/kg, *i.p.*, myricetin reduced menthol-induced nociception by 95% with a mean ID_50_ of 2.4 mg/kg. Moreover, it also reduced menthol-induced mechanical allodynia at 30 and 100 mg/kg, *i.p.*

### 3.10. Activity Against Bone-Related Disorders

Parathyroid hormone-induced osteoclast-like cell formation in mouse marrow culture was inhibited *in vitro* by myricetin (10^−8^ M). It also prevented a PTH-induced decrease in diaphyseal calcium content at a concentration of 10^−6^ M [139]. Hsu and coworkers [140] reported that myricetin increases BMP-2 synthesis, resulting in the subsequent activation of SMAD1/5/8 and p38 MAPK. This activity is perhaps related to the induction of osteoblast maturation and differentiation, followed by an increase in bone mass. The compound was shown to stimulate osteoblast differentiation at various stages, from maturation to terminal differentiation. Induction of differentiation by myricetin was found to be associated with increased bone morphogenetic protein-2 (BMP-2) production, and increased activation of SMAD1/5/8 and p38 MAPK.

### 3.11. Activity against CNS Disorders

Much research on myricetin has focused on its value as an agent to mitigate neurodegenerative diseases with many studies focusing on the interaction of myricetin and specific brain receptors. The compound is known to have protective effects against the progression of Parkinson’s disease (PD) and Alzheimer’s disease (AD). The latter is distinguished mainly by neuronal loss and is characterised by two typical lesions, *i.e.*, neurofibrillary tangles and plaques comprising β-amyloid [141].

The protective effects of myricetin stem from the effect of the compound against specific proteins, known as tau proteins, which are abundant in the distal portions of axons and serve to provide flexibility and stability to microtubules [142]. Pathologies of the nervous system, such as AD and PD can develop when tau proteins become defective and are subsequently unable to sufficiently stabilize microtubules. These tau proteins impart stability to the microtubules through isoforms and phosphorylation. However, hyperphosphorylation of tau proteins causes entanglement of the helical and straight neurofilaments, thereby contributing to the progression of AD [143]. All six isoforms of tau proteins are commonly found in their hyperphosphorylated form in filaments of brains affected by AD. Abnormal aggregates containing large amounts of tau proteins have also been associated with other neurodegenerative diseases. Microtubule-associated protein tau, has therefore been identified as a target protein for AD [2]. Myricetin was found to produce an anti-tau effect at a concentration of 50 µM in HeLa-C3 cells.

A second-mode of action that has been investigated widely is the ability of myricetin to block Alzheimer associated β-amyloid fibril formation [144]. Some oxidative effects towards the Met35 residue in amyloid-β-(Aβ) peptides, involved in AD, were brought about by myricetin through maintenance of the monomer levels and by interfering with the formation of ordered Aβ42 aggregates. Supporting evidence that the molecule has the ability to interfere with β-amyloid fibril formation was obtained in a docking study [145], thereby indicating a strong possibility of anti-amyloidogenic activity. Quantitative data on the effects of myricetin on β-amyloid fibrils (fAβ) indicated that at 37 °C and pH 7.5 the compound is able to inhibit their formation, extension and destabilization from fresh Aβ (1-40)- and Aβ (1-42)-peptides, with corresponding EC_50_ values of 0.1–1 μM. At a concentration of 300 nM, the compound potentially reduced β-amyloid-induced cell injury (Aβ1-42, 1 μM) of rat cortical neurons, whereas, at 10 μM, it prevented structural changes from a random coil to a β-sheet-rich structure in Aβ(1-42). The compound was also able to induce a reduction in Aβ(1-40) and Aβ(1-42) levels [146].

The excessive release of glutamate is a serious component in the neuropathology of acute and chronic brain disorders. Interestingly, myricetin inhibited the glutamate release from cerebrocortical synaptosomes by attenuating voltage-dependent Ca^2+^ entry [147]. It potentially inhibited 4-AP-evoked glutamate release in the nerve terminals of the rat cerebral cortex which was prevented by chelating extracellular Ca^2+^ ions and vesicular transporter inhibitor bafilomycin A1. It reduced 4-AP-induced increases in cytosolic free Ca^2+^ concentration without altering synaptosomal membrane potential. This compound was found to be a potent anti-neurodegenerative agent, able to inhibit glutamate-induced excitotoxicity. In addition, it was also reported to protect neurons in the rat cerebral cortex *in vitro,* by reducing glutamate-induced intracellular Ca^2+^ overload, inhibiting ROS production and reducing glutamate-induced activation of caspase-3 [148]. Such an effect could be very important to be considered as glutamate levels have been found to increase after *in vivo* and *in vitro* exposure to soluble forms of amyloid beta [149].

Further evidence that the compound acts as an anti-fibrillogenic agent was furnished by Ono and Yamada [150]. They found that the compound inhibits the formation of α-synuclein (αS) fibrils and destabilises them in the brain, a key step in the prevention of Lewy body disease, multiple system atrophy and AD. The compound was reported to also inhibit the aggregation of αS into oligomers, a process involved in the pathogenesis of PD, by 10%, with a corresponding IC_50_ value of 3.57 µM using DMSO as the control [151]. The study aimed at elucidating the mode of action revealed that the aromatic rings and vicinal hydroxy groups in the structure are most probably responsible for the activity. It is noteworthy that the activity of catechol *O*-methyltransferase (COMT) was significantly inhibited *in vitro* by myricetin, with an inhibition kinetic parameter (Ki) value of 0.2 μM [152]. This enzyme is key to the metabolism of levodopa, which is used to treat PD by increasing dopamine concentrations in the brain. However, Jimenez and coworkers [153] found that myricetin, even at concentrations of 50 and 100 μM, displayed no neuroprotective effects towards rotenone-induced apoptosis in SH-SY5Y cells, which contribute to the etiology of PD. In contrast, N-acetylcysteine (100 μM) displayed a potent protective activity. These findings reveal that there is still much to be learnt concerning the beneficial effects of myricetin.

The ability of myricetin to act as a neuroprotective agent against A2E and light damage in photoreceptor cells has been reported. It inhibited damage to blue light-induced photoreceptors with an EC_50_ of 9 μM based on green nucleic acid stain, whereas, at 40 μM, it protected all of the exposed photoreceptors against blue-light-mediated damage [154]. The compound afforded protection against A2E-induced photoreceptors and bipolar cell death with a corresponding EC_50_ value of 2 μM. Myricetin also mitigated oxidative stress, increased the activity of Na^+^ and K^+^-ATPase, and controlled the expression of extracellular signal-regulated kinase-cyclic AMP response element binding protein signalling pathway against *d*-galactose-induced cognitive impairment in mice [155]. It also inhibited sodium dithionite-induced ischemic injury in cultured rat cortical neurons by 33.9% at 1 × 10^−5^ g/mL [156]. Myricetin (30 and 100 mg/kg, p.o.) displayed anxiolytic activity in various mouse behavioral models [157]. It effectively reduced lithium-induced head twitches and also antagonized *m*-chlorophenylpiperazine-aroused anxiety. The study revealed that the mode of action is linked to the modification of serotonin transmission by myricetin. By using an automated sequential injection spectrophotometric system, Moonrungsee and coworkers [158] demonstrated the ability of myricetin to inhibit tyramine oxidase, an enzyme that plays a vital role in the inactivation of neurotransmitters, as reflected by the IC_50_ value of 0.04 mM obtained for the compound.

The toxicity of 6-hydroxydopamine was found to be reduced by myricetin, by decreasing the dopamine content in the *substantia nigra*-striatum system [159]. The compound also prevented a 6-hydroxydopamine-induced decrease of tyrosine hydroxylase positive neurons and tyrosine hydroxylase mRNA expression in the same region of the brain. The compound was also reported to decrease methyl mercury-induced mouse brain mitochondrial dysfunction and oxidative stress *in vitro* [160]. It also blocked ROS formation and lipid peroxidation completely and partially prevented glutathione depletion. Myricetin was found to exert a neuroprotective effect towards 1-methyl-4-phenylpyridinium (MPP+)-induced MES23.5 cells, by reducing cell loss and nuclear condensation. It also suppressed the production of intracellular ROS, restored the mitochondrial transmembrane potential, increased the Bcl-2/Bax ratio and decreased caspase-3 activation induced by MPP+. Moreover, it decreased the phosphorylation of MAPK kinase-4 and JNK caused by MPP+ [161].

It was reported that myricetin promotes GABAergic activity in the neurons of the hypothalamic paraventricular nucleus (PVN) by increasing the decay time and frequency of the inhibitory currents mediated by the GABAA receptor. The compound was able to increase the Ca^2+^-current and intracellular Ca^2+^ concentration, respectively, via T- and L-type Ca^2+^ channels in rat PVN neurons and hypothalamic primary culture neurons. It also increased phosphorylation of Ca^2+^/calmodulin-stimulated protein kinase II incubation in a primary culture of rat hypothalamic neurons in PC-12 cells [162]. Myricetin was found to inhibit neurotransmitter release from neuronal PC12 cells by interfering with SNARE complex formation. It caused muscle paralysis by inhibiting acetylcholine release at the neuromuscular junction. However, the effect of myricetin was shorter and less effective when compared with that of *Clostridium botulinum* neurotoxin (BoNT/A) [163]. It reduced the action potential frequency in type-I PVN neurons in hypothalamic brain slices of rats. Ma and Liu [164] reported that myricetin was able to enhance K^+^ currents by shifting the voltage-dependence of activation of potassium currents to more negative potentials by 6 mV.

### 3.12. Hepatoprotective and Hypouricemic Activities

These activities have been demonstrated *in vivo* using mice models. Myricetin exerted antigenotoxic and hepatoprotective effects against pyrogallol-induced toxicity in mice [165]. It was found to promote the restoration of hepatic function by reducing pyrogallol-induced elevation of the serum enzymes AST, ALT, ALP and in total bilirubin. The compound also reduced DNA damage in the liver.

Myricetin exhibited activity in potassium oxonate-induced hyperuricemic mice following oral administration of 50 and 100 mg/kg for 3 days. It was found to reduce liver uric acid levels, in addition to inhibiting liver xanthine oxidase activity in mice. Studies on the mechanism of action suggest that the hydroxylated planar structure of the molecule is mainly responsible for the hypouricemic activity [166].

### 3.13. Activity against Cardiovascular Diseases

Myricetin (100 μM) displayed a vasculoprotective effect through transcriptional changes in human umbilical vein endothelial cells, as determined by microarray gene expression profiling [167]. Simultaneously, it also altered vascular disease-related genes, including HIRA, HDAC9, HIF1A, and RTN3. The compound at a concentration of 50 μM increased the KCl-induced intracellular Ca^2+^ concentration in cardiomyocytes of rats in Tyrode’s solution [168]. These results revealed that myricetin inhibits the voltage-dependent Ca^2+^ channel, which is thought to protect cardiomyocytes. Furthermore, Scarabelli and coworkers [169] found that myricetin, under hypoxic conditions, is able to decrease the rate of both necrotic and apoptotic cell death in neonatal cardiomyocytes. An *ex vivo* study revealed that myricetin infusion for 1 h, prior to onset of ischemia and during reperfusion, potentially reduced the intract size in the Langendorff perfused rat heart [170]. No cytotoxicity, even at a concentration of 30 μM, was observed in cultured neonatal rat cardiomyocytes. However, the compound displayed a protective effect towards the H_2_O_2_-induced apoptosis of cardiomyocytes at this concentration, by inhibiting the activation of caspase-3 protein, up-regulating the expression of Bcl-2 and down-regulating the expression of Bax. At a concentration of 50 μM, it partially inhibited the KCl-induced vasorelaxant effect in intact rings by inhibiting of Na^+^/K^+^-ATPase activity and activating protein kinases [171]. It also induced an endothelium-dependent contractile response, which was increased in the presence of PMA and reduced by staurosporine. Oral administration of 100 and 300 mg/kg doses to Wistar rats resulted in a reduction in heart rate and the levels of cardiac marker enzymes (lactate dehydrogenase, creatine kinase, aspartate aminotransferase, SOD and CAT), as well as changes in vascular reactivity and electrocardiographic patterns caused by isoproterenol [172]. Lian and coworkers [173] suggested that myricetin could act as an active agent to prevent atherosclerosis, while Angelone and co-workers [174] described the compound as a potent cardio-active agent that is able to protect the heart in the presence of cardiovascular diseases. At a concentration of 20 μM, it inhibited CD36 cell surface protein and mRNA expression in U937-derived macrophages. Myricetin elicited coronary dilation, without affecting contractility and relaxation of isolated and Langendorff perfused rat hearts. An *in vivo* study by Bhatia and coworkers [175] indicated that it exerts lipid lowering activity in Triton-treated hyperlipidemic rats and could be helpful in treating hyperlipidemia and related cardiovascular diseases.

### 3.14. Activity against Eye Disorders

Myricetin, a strong aldose reductase inhibitor, exhibited anticataract activity in galactosemic rats at a concentration of 1%. It delayed both the onset and progression of cataract development in the eyes. Moreover, it was found to be safe for topical administration [176]. An *in vivo* study by Hodges and coworkers [177] revealed that myricetin lowered the intraocular pressure below that of the control in normotensive rabbits at a dose of 1 mg/kg, *i.v.* This finding indicates the potential role of the compound for the treatment of glaucoma. In an *in vitro* study, myricetin at various concentrations, *i.e.*, 10, 20, 50 and 100 µM, decreased human retinal pigment epithelial cell proliferation and migration, as well as the secretion of vascular endothelial growth factor in culture [178]. At low concentrations, it was found to reduce gene expression of vascular endothelial growth factor, while at high concentrations (>100 µM) it elevated gene expression. The compound also affected a decrease in cell viability by activating cellular necrosis at higher doses. It caused caspase-3 independent retinal pigment epithelial cell necrosis mediated by free radical generation and activation of calpain and phospholipase A2.

### 3.15. Antidiabetic and Anti-Obesity Activities

Myricetin has been proven to have potential for the management of non-insulin-dependent diabetes, by stimulating the uptake of glucose without functional insulin receptors [179]. The compound was found to enhance the stimulatory activity of insulin as reflected by the EC_50_ value of 65 μM obtained. It also stimulated d-glucose and d-3-*O*-methylglucose uptake in rat adipocytes. A further *in vivo* study by the same researchers [180] demonstrated that myricetin, intraperitoneally administered to streptozotocin-induced diabetic rats at a dose of 3 mg/kg/12 h for 2 days, resulted in a 50% decrease in hyperglycemia and an increase in hepatic glycogen and glucose-6-phosphate content. It was further suggested that myricetin has antidiabetic activity without any serious hepatotoxicity. This activity was linked to the effect of the molecule on glycogen metabolism. It inhibited the aggregation of islet amyloid polypeptide (IAPP), which is recognised to play a major role in the death of pancreatic β-islet cells in type II diabetes. Myricetin prevented thioflavin T binding and fibre formation conducive to forming IAPP aggregates. It also slowed the *in vivo* aggregation of IAPP-EGFP and protected living mammalian cells from the toxic effects of IAPP [181].

The compound was found to decrease high glucose plasma levels and improve insulin resistance by enhancing β-endorphin production in fructose-induced insulin-resistant rats intravenously dosed with 1 mg/kg, thrice a day for 14 days. It also affected the phosphorylation of insulin receptor, insulin receptor substrate-1, Akt, Akt substrate of 160 kDa and glucose-transporter subtype-4 translocation [182,183]. A further study [184] revealed that intravenous administration of myricetin at 1 mg/kg, thrice a day for one week, to obese Zucker rats, decreased the concentration of plasma glucose and the glucose-insulin index value. This study also suggested that myricetin could reduce insulin sensitivity through increased post-receptor insulin signalling, mediated by enhancements in IRS-1-associated PI3-kinase and GLUT 4 activity in the muscles of experimental rats.

Ding and coworkers [185] found that the compound improves low-dose insulin-stimulated glucose uptake in the hyperinsulinemic state in the skeletal muscle cell line C2C12 myotubes. The compound displayed cytoprotective effects against cytokine-induced cell death in insulin-secreting RIN-m5f β cells at 10 and 20 μM by increasing cell viability and decreasing cell apoptosis induced by cytokine. It reduced cytokine-mediated increased levels of NFκB, decreased inhibitor κB α levels, stimulated NO accumulation, increased cytochrome c release from mitochondria and induced ROS generation. Myricetin was reported to increase cell survival, alkaline phosphatase activity, collagen, osteocalcin, osteoprotegerin, and calcium deposition and decreased cellular malondialdehyde, protein carbonyl and advanced the oxidation protein product contents of osteoblastic MC3T3-E1 cells induced by 2-deoxy-d-ribose-induced. These effects of myricetin suggest that it could reduce oxidative injury in diabetes-related bone diseases [186].

An *in vivo* study by Ozcan and coworkers [187] suggested that myricetin could be used for the treatment of diabetic nephropathy. In this study, intraperitoneal administration of myricetin at 6 mg/kg/day to streptozotocin-induced diabetic rats reduced glomerulosclerosis, blood urea nitrogen, urinary volume and protein excretion. It also restored decreased renal activities of glutathione peroxidase and increased the activity of xanthine oxidase in diabetic rats. A mechanism-based study by Liu and coworkers [188] indicated that the intravenous administration of myricetin at 1 mg/kg, thrice a day for 3 days, decreased blood glucose levels and increased blood β-endorphin-like immunoreactivity in streptozotocin-induced diabetic rats. The compound’s blood glucose-lowering effect in insulin-deficient rats is mediated through activation of opioid μ-receptors of peripheral tissues in response to increased β-endorphin secretion. Interestingly, intraperitoneal administration of myricetin at 1 mg/kg b.w. induced antihyperglycemic and renal protective activities in streptozotocin-cadmium-induced diabetic nephrotoxic rats [189]. These effects were attributed to a reduction in the levels of plasma glucose and glycosylated haemoglobin and an increase in the levels of plasma insulin, body weight and total haemoglobin.

Pretreatment of pancreatic RIN-m5f β cells with myricetin at 20 μM protected against cytokine-induced cell death. It also decreased basal insulin secretion and reduced glucose-stimulated insulin secretion in cytokine-treated RIN-m5f cells. The study indicated that the compound could restrict cell dysfunction in cytokine-induced RIN-m5F cells via the Wnt signal pathway [190]. A mechanism-based study suggested that myricetin, a human α-amylase inhibitor, binds at the active site, subsequently interacting directly with the catalytic residues and thereby reduces the normal conformational flexibility of the adjacent substrate binding cleft [191].

Myricetin was found to stimulate hepatocellular cholesterol biosynthesis in rat hepatocytes at a concentration of 10 μM. However, at higher concentrations, it was found to inhibit cholesterol biosynthesis in rat hepatocytes, but to stimulate the production in HepG2 cells [192]. Chang and coworkers [193] demonstrated that myricetin exerts strong anti-obesity and anti-hyperlipidaemic activities. It was found to decrease the intracellular accumulation of triglycerides in 3T3-L1 adipocytes in high-fat diet-fed rats at an oral dose of 300 mg/kg/day over eight weeks. This effects of myricetin in reducing body weight, visceral fat-pad weights and plasma lipid levels of fat-fed rats were found similar to those of fenofibrate at 100 mg/kg/day. The hepatic triglyceride and cholesterol contents were also decreased by myricetin treatment. The reduction of body weight gain and fat accumulation was attributed to accelerated fatty acid oxidation in rat livers.

### 3.16. Antimicrobial Activity

Myricetin displayed antibacterial and antiviral activities against several organisms. A noteworthy activity (20 µg/mL) was established against the Gram-negative anaerobic periodontal oral pathogens, *Porphyromonas gingivalis* and *Prevotella intermedia* [194]. Poor activity was reported against *Streptococcus mutans* and *Actinomyces viscosus* with MIC values of 2500 and 1250 µg/mL, respectively. Although strong activity was found against *Pseudomonas aeruginosa* (MIC 1.5 μg/mL), myricetin exhibited synergistic effects with sulfamethoxazole against three strains (PA01, DB5218 and DR3062) of the bacterium [195]. The compound displayed poor activity against *Klebsiella pneumoniae* (MIC_50_ 128 mg/mL), but in combination with amoxicillin/clavulanate, ampicillin/sulbactam and cefoxitin, strong synergy was observed at a concentration of 32 µg/mL [196]. D’Souza and coworkers [197] found myricetin to be strongly active against *K. pneumoniae, P. mirabilis, P. aeruginosa* and *Shigella flexineri* at a concentration of 30 µg/mL.

Myricetin was found to inhibit *E. coli* DnaB helicase with an IC_50_ value of 11.3 μM. This essential enzyme plays a key role in the replication and elongation of DNA [198]. However, the compound exhibited poor inhibitory activity against *E. coli* primase; the activity was 60-fold weaker than against DnaB helicase. It also inhibited the growth of methicillin-resistant *Staphylococcus aureus,* multidrug-resistant *Burkholderia cepacia* and vancomycin-resistant *Enterococci*. Results from a radiolabel incorporation assay revealed that myricetin inhibits protein synthesis of *B. cepacia* [199]. It also exhibits a potent activity against recombinant sortase A and B obtained from *S. aureus* with IC_50_ values of 44.03 and 36.89 µM, respectively, and a corresponding MIC value above 300 µM [200]. At a concentration of 0.5 mg/mL, myricetin produced significant zones of inhibition, ranging from 13.4 mm to 19.2 mm, against *B. subtilis, Corynebacterium diphtheria, C. diphtheriticum, Micrococcus lysodiecticus, S. aureus, S. epidermidis, S. saprophyticus, Enterococcus faecalis, E. faecium, Streptococcus pneumonia, S. pyogenes, E. coli, K. pneumonia, P. mirabilis, P. aeruginosa, S. typhi, S. paratyphi, S. dysenteriae, S. sonneie* and *S. flexneriae*, but the potency was higher against Gram-positive bacteria [201].

Antitubercular activity was recorded (MIC 50 µg/mL) after exposing *Mycobacterium tuberculosis* to myricetin [202]. A structure-activity relationship analysis left no doubt that the hydroxy groups in the structure are responsible for this activity.

Myricetin was found to be a strong inhibitor of reverse transcriptase from Rauscher murine leukemia virus (RLV) and human immunodeficiency virus (HIV). At a concentration of 1 and 2 µg/mL, it completely inhibited the activities of RLV and HIV reverse transcriptase, with a Ki value of 0.08 µM recorded for the latter. The compound also inhibited DNA polymerase α and DNA polymerase I [203]. In an *in silico* experiment, it demonstrated anti-HIV activity with an electron-ion interaction potential and average quasi valence numbers of 0.110 and 3.576, respectively [204]. However, an *in vitro* study evaluating myricetin by Yang and coworkers [205] yielded no activity against HIV-1. Chu and coworkers [206] reported that the compound is able to inhibit Moloney murine leukemia virus reverse transcriptase activity. Their structure-activity relationship study suggested that the hydroxy groups at positions C3 and C4 increased the activity. The compound was reported to display activity against the SARS-coronavirus, a causative agent for severe acute respiratory syndrome, and inhibited the coronavirus helicase protein by affecting the ATPase activity *in vitro* at an IC_50_ value of 2.71 µM. However, it was found ineffective against the hepatitis C virus helicase. A toxicity study suggested that myricetin does not exert cytotoxicity towards normal breast epithelial MCF10A cells, hence, researchers concluded that the compound is safe for further *in vivo* studies [207,208].

### 3.17. Miscellaneous Activities

Myricetin could be used to treat circadian rhythm disorders by changing the circadian rhythm of serum melatonin and locomotor activity. It was found to inhibit the activity of arylalkylamine-N-acetyltransferase, a rate-limiting enzyme in the melatonin biosynthetic pathway, which catalyses the conversion of serotonin to N-acetylserotonin, and also to decrease the nocturnal serum melatonin levels in rats [209].

At a concentration of 100 μM, it inhibited ATP-dependent Ca^2+^ uptake by rat liver plasma membrane vesicles by more than 20%. At the same concentration, it inhibited K^+^-dependent *p*-nitrophenyl phosphatase by 83%, whereas it did not exert any effect on 5′-nucleotidase, alkaline phosphatase and Ca^2+^-activated ATPase. Myricetin (52 μM) lowered the initial rate of ^45^Ca uptake by 50% after pre-incubating for 10 min. The mechanism of activity suggested that lipid solubility and hydroxylation at positions 5, 7, 3′, 4′ in the structure improved the ability to inhibit Ca^2+^ uptake [210].

Myricetin (0.03 mM) was found to inhibit lipoxygenase activity by 91% in liver cytosol of rats fed oxidized palm oil [211]. It exhibited protective effects against the genotoxicity of a hormonal steroid, 17β-estradiol in peripheral blood human lymphocyte culture at a concentration of 10 μM [212]. It decreased the production of oxygen-glucose deprivation-induced free radical, responsible for swelling of C6 glial cells. The compound was found to attenuate increased levels of intracellular calcium, the main characteristic of ischemic injury to cells, at various concentrations, *i.e.*, 100 pM, 1 nM and 10 nM [213].

Estrogens, the primary female sex hormones, play a vital role in both menstrual and estrous reproductive cycles. Oral administration of myricetin at 100 mg/kg/day, caused estrogenic activity by increasing the uterus weight and height in immature Wistar albino rats when compared to that of controls (ethinyl estradiol, ethinyl estradiol + tamoxifen and genistein) [214].

## 4. Toxicity Studies

Myricetin has been exhaustively studied in all types of *in vitro* and *in vivo* studies. Very few of these studies have raised concerns with regards to adverse effects. Intraperitoneal administration of myricetin at a dose of 1 000 mg/kg b.w. to mice did not reveal any toxic effects or fatalities [163]. The compound did not cause any toxicity at doses above 100 mg/kg (LD_50_ value) in zebrafish larvae induced by UVB-generated ROS [215]. A study by Kim and coworkers [216] suggested that myricetin is not cytotoxic towards human umbilical vein endothelial cells (HUVECs). The hydroxy groups on the B-ring were linked to the protective effect. An LD_50_ value of 100 μM was established for myricetin. At 50 μM, it suppressed HUVEC tubular structure formation stimulated by vascular endothelial growth factor (VEGF) by 47%.

Some researchers have suggested that myricetin may be toxic towards biological cells. Canada and coworkers [217] reported that the compound, at 450 μM, causes cellular damage to isolated guinea pig enterocytes. The cellular viability was reduced by as much as 60% and lactic dehydrogenase leakage was increased by 41%. Superoxide is produced by autoxidation and thought to be responsible for the toxicity of the compound, since the radical may produce intestinal injury. A few studies have indicated that myricetin could exert pro-oxidant effects at high concentrations in ascorbic acid-free systems with the formation of the Fe-EDTA complex [53,54].

## 5. Recommendations and Concluding Remarks

Myricetin is a key ingredient in many foods and is used as a food additive as a result of its anti-oxidant activity and ability to protect lipids against oxidative damage. Available literature portrays the compound as a wonder nutraceuticals and there is no doubt that the molecule holds potential to protect against life threatening diseases, including cancer. The most noteworthy of the biological activities is the protective effect of the compound towards diseases affecting the elderly, such as PD and AD. Although, myricetin alone displays a variety of activities, it seems that its activity may be considerable enhanced through additive or synergistic interactions when in combination with other bioactive compounds [196]. Various researchers have demonstrated its protective nature towards skin aging, suggesting that the compound could be used in cosmetic preparations. In addition, its periphery analgesic effect by enhancing calcium depended potassium channel current and inhibiting excitability of small neurons of dorsal root ganglion in *in vivo* models supports its role in pain and inflammation [218]. Hence, based on the results from various *in vivo* studies, myricetin can be developed as an anti-inflammatory and analgesic agent in near future.

However, it has been reported that carbohydrates, DNA and other non-lipid elements in food are degraded by this molecule [219]. Hakkinen and coworkers [220] found that myricetin is susceptible to degradation, since it is labile at high temperatures and is sensitive to certain pH conditions. Food processing and storage is also known to affect the available concentration. These factors should be taken into account before myricetin is used in particular formulations [9].

The compound has been found to be non-toxic in several *in vivo* models, although Canada and coworkers [217] reported a degree of toxicity to intestinal cells. This molecule also exerts pro-oxidant effects at higher concentrations [54]. These findings suggest that more toxicity studies should be undertaken before myricetin is included in nutraceuticals and cosmetic preparations.

## Abbreviations

The following abbreviations are used in this manuscript:

BMP-2: Bone morphogenetic protein-2

DCs: dendritic cells

EGF: epidermal growth factor

HGF: Human gingival fibroblasts

GSH: intracellular reduced glutathione

Io: ionomycin

JNK: Jun NH2-terminal kinase

LPS: lipopolysaccharide

MMP: matrix metalloproteinase

MAPK: mitogen-activated protein kinase

NADH: nicotinamide adenine dinucleotide hydride

OVA: ovalbumin

p38: p38 mitogen-activated protein kinase

PVN: paraventricular nucleus

PD: Parkinson’s disease

PMA: phorbol 12-myristate 13-acetate

PMACI: phorbol-12-myristate-13-acetate and calcium ionophore A23187

PGE2: prostaglandin E2

PKC: protein kinase C

ROS: Reactive oxygen species

RANKL: receptor activator of NF-κB ligand

TBARS: thiobarbituric acid substances

TNF-α: tumor necrosis factor-α

IL-1β: interleukin-1β

TRAIL: tumour necrosis factor-related apoptosis-inducing ligand

TPA: 12-*O*-tetradecanoylphorbol-13-acetate

MPP+: 1-methyl-4-phenylpyridinium

DPPH: 2,2-diphenyl-1-picrylhydrazyl.
